# Ocular Coloboma With Choroidal Neovascular Membrane: A Case Report

**DOI:** 10.7759/cureus.19521

**Published:** 2021-11-13

**Authors:** Jluwi Almasaud, Sara A Aledaili, Reem S Alshammari

**Affiliations:** 1 Ophthalmology, King Khaled Hospital, Hail, SAU; 2 Medicine, University of Hail College of Medicine, Hail, SAU; 3 Ophthalmology, University of Hail College of Medicine, Hail, SAU

**Keywords:** congenital, choroidal neovascular membrane, ophthalmology, iris, ocular coloboma

## Abstract

Ocular coloboma (OC) is a rare congenital anomaly and a product of a defect in embryogenesis. It is the result of fetal fissure closure error that ends with a persistent cleft. Colobomas are generally accompanied by visual loss. In this article, we present a case of bilateral iris, disc, and retina coloboma that was managed with an anti-vascular endothelial growth factor (ranibizumab), and as a result, caused regression of the choroidal neovascular membranes and improved the patient's visual acuity. However, The patient will need lifelong follow-up to catch any retinochoroidal changes or development of cataract or glaucoma.

## Introduction

Ocular coloboma (OC) is a congenital anomaly represented by missing pieces of tissue in the eye structure that appear as notches or gaps in the iris, retina, choroid, or optic nerve. Eye development starts on the 20th day of embryo life when a pair of shallow grooves appear on the sides of the invaginating forebrain [1]. Ocular coloboma is the final product of an error occurring in the closure of the fetal fissure. Hence, it ends with a persistent cleft; the normal process takes place from the fifth to sixth weeks of pregnancy [2]. Generally, colobomas are associated with visual loss, which may need management and intervention besides amblyopia management [3]. Therefore, recognition of congenital anomalies of the eye can improve parents' understanding as well as genetic counseling. In this article, we present a case of bilateral iris, disc, and retina coloboma. The article was presented as a poster at the 2nd Global Congress on Medical & Clinical Case Reports in Dubai, United Arab Emirates, on November 19, 2018.

## Case presentation

**Figure 1:**
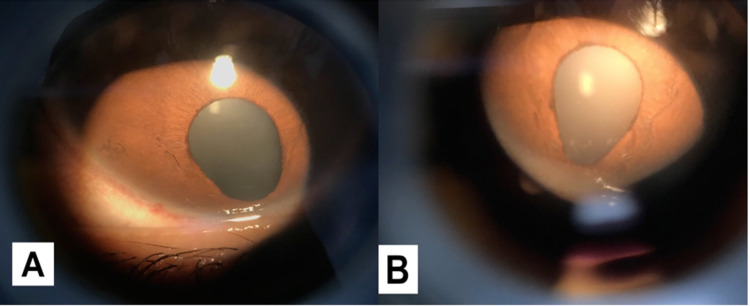
(A) Right eye iris coloboma. (B) Left eye iris coloboma.

A 20-year-old male was referred from a remote village to an Ophthalmology clinic with a painless as well as progressive decrease of vision in the right eye for the past two months. The decrease of vision in the right eye was gradual, with no pain nor history of ocular trauma or surgeries. His left eye was amblyopic. The patient had no medical history or any significant family history. On ocular examination, his unassisted visual acuity was 20/200 in his right eye and hand motion in his left eye. The intraocular pressure was 18 mmHg bilaterally. On inspection, there was exotropia of the left eye. Next, the slit lamp evaluation of the anterior segment was within normal limits except for iris coloboma in the inferior nasal quadrant of each eye (Figure 1).

**Figure 2:**
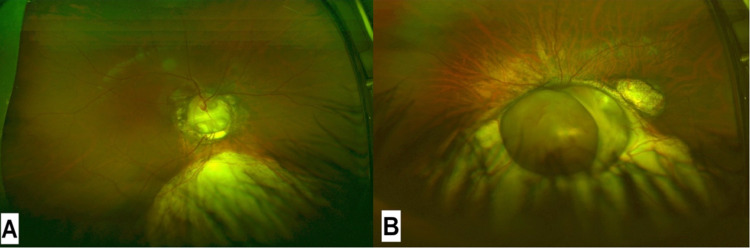
(A) Right eye optic disc inferonasal retina coloboma. (B) Left eye optic disc retina coloboma.

**Figure 3:**
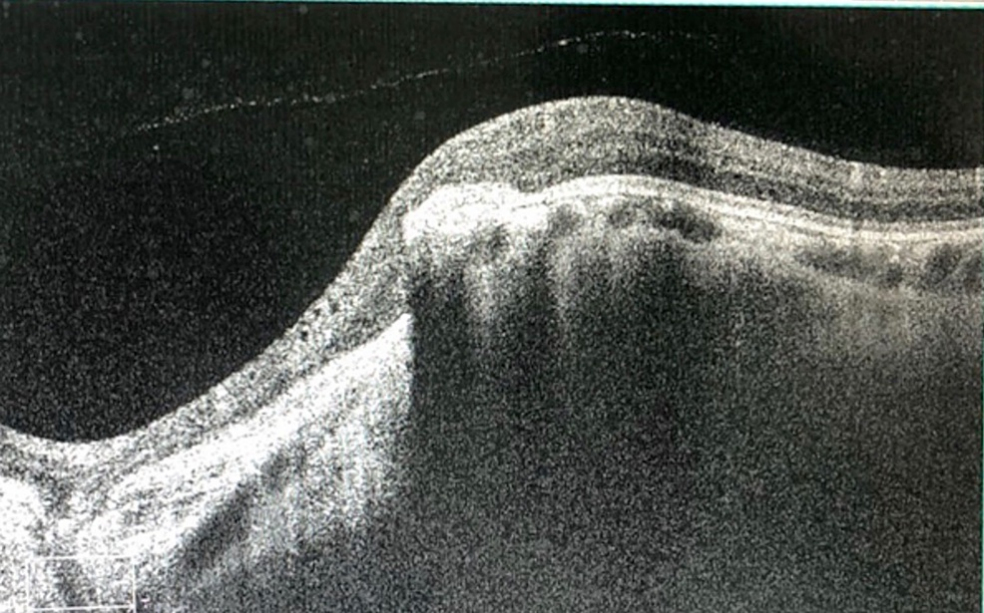
Right eye OCT. OCT: optical coherence tomography

Dilated fundus examination showed optic disc and retinochoroidal coloboma in both eyes (Figure 2) with juxtapapillary choroidal neovascularization in the right eye. Furthermore, we performed optical coherence tomography (OCT), which confirmed the presence of choroidal neovascular membrane (CNVM) (Figure 3).

## Discussion

As it is a rare congenital anomaly, ocular coloboma (OC) has a prevalence that is around 0.14% of the general population [4]. It is initiated by imperfect embryogenesis, which leads to failed closure of the embryonic fissure during the first trimester of fetal life [5]. Worldwide, the incidence of the condition is seven for every 10,000 live births [1]. In this report, we presented a patient with bilateral iris, optic disc, retinochoroidal coloboma. Unfortunately, in Saudi Arabia, no records exist of the incidence of OC. The occurrence of sporadic, unilateral, or bilateral coloboma varies with genetic and non-genetic factors; it also could be related to some syndromes such as coloboma, heart anomaly, choanal atresia, retardation, genital and ear anomalies (CHARGE) syndrome [6]. Studies suggest that the usage of some drugs during pregnancy could be linked with OC [7,8].

Here we described a case of OC associated with optic disc, retina, choroid, and iris coloboma that we consider unlikely to be inherited because neither one of the parents or siblings of the patients had the same condition. And it is less often that an autosomal dominant inheritance may occur [9]. Chromosomal analysis is needed for a patient with OC, but it was not possible in our case due to non-availability at our facilities.

Our patient presented with bilateral iris, optic disc, and retinochoroidal coloboma, which is consistent with previous studies that suggest that 50% percent of patients with OC have bilateral disease [10]. He also had right choroidal neovascularization in the inferior nasal area that caused the decrease of vision. In such cases, the treatment plan will include either anti-vascular endothelial growth factor (anti-VEGF), focal laser photocoagulation, or photodynamic therapy. According to the literature, it is usually preferred to start with focal laser photocoagulation [11], but regarding our patient who had the CNVM in juxtapapillary area, we decided to treat him with anti-VEGF to avoid the potential risk of iatrogenic vision loss. The treatment with anti-VEGF has resulted in regression of the CNVM, hence improving the patient's visual acuity. Furthermore, we plan to continue the patient on more anti-VEGF until reaching complete regression of the CNVM and routine follow-up in the future. 

## Conclusions

To the best of our knowledge, the presented case is the first case of coloboma reported in the northern region of Saudi Arabia. We recommend early recognition of such cases, which will improve parents' understanding as well as genetic counseling. Furthermore, we also recommend lifelong follow-up for coloboma cases to catch any retinochoroidal changes or development of cataract and glaucoma. Correspondingly, more studies are needed to better understand ocular coloboma's prevalence, causes, and management. 
